# The Role of Direct-Acting Antivirals (DAAs) in Hepatitis C Virus-Associated Lymphoproliferative Disorders

**DOI:** 10.3390/cancers18152501

**Published:** 2026-08-04

**Authors:** Cesare Mazzaro, Riccardo Bomben, Laura Gragnani, Marcella Visentini, Paolo Agostinis, Silvia Marri, Anna Linda Zignego, Valter Gattei

**Affiliations:** 1 Clinical and Experimental Onco-Haematology Unit, Centro di Riferimento Oncologico di Aviano (CRO) IRCCS, 33081 Aviano, Italy; vgattei@cro.it; 2 Department of Translational Research and New Surgical and Medical Technologies, University of Pisa, 56126 Pisa, Italy; laura.gragnani@unipi.it; 3 Laboratory Affiliated to Istituto Pasteur Italia-Fondazione Cenci Bolognetti, Department of Translational and Precision Medicine, Sapienza University of Rome, 00185 Rome, Italy; marcella.visentini@uniroma1.it; 4 Department of Internal Medicine, Hospital of Tolmezzo, 33028 Tolmezzo, Italy; paolo.agostinis@asufc.sanita.fvg.it; 5 Department of Experimental and Clinical Medicine, University of Florence, 50134 Florence, Italy; silvia.marri@unifi.it (S.M.); annalinda.zignego@unifi.it (A.L.Z.)

**Keywords:** hepatitis C virus, extrahepatic manifestations, mixed cryoglobulinemia, cryoglobulinemic vasculitis, direct-acting antiviral agents, B-cell non-Hodgkin lymphoma, rituximab

## Abstract

Hepatitis C virus (HCV) infection is a major cause of chronic hepatitis, affecting approximately 50 million people worldwide. An estimated 15–30% of individuals with chronic HCV infection progress to cirrhosis, which may subsequently develop into hepatocellular carcinoma. Beyond liver disease, HCV is associated with a broad spectrum of extrahepatic manifestations, particularly mixed cryoglobulinemia (MC) and B-cell non-Hodgkin lymphoma (B-NHL). Persistent viral infection induces chronic antigenic stimulation of B lymphocytes, a key pathogenic mechanism underlying the progression from MC to overt B-NHL. These observations have important therapeutic implications and support the use of antiviral therapy as a cornerstone of treatment. The efficacy of direct-acting antivirals (DAAs) has been well established in patients with HCV-related MC and cryoglobulinemic vasculitis. Several studies have shown that DAAs achieve sustained virologic response rates exceeding 90%, often approaching 100% in contemporary cohorts. Viral eradication is frequently associated with clinical and immunological improvement, as well as regression of cryoglobulinemia. Encouraging outcomes have also been reported in patients with HCV-associated indolent B-NHL, particularly marginal zone lymphoma, although confirmation in larger studies with longer follow-up is needed. In patients with HCV-positive aggressive lymphomas, DAAs have been safely administered in combination with immunochemotherapy, yielding promising results. This review summarizes the current evidence on HCV-associated MC and B-NHL and discusses the impact of DAA therapy on the clinical course and management of these disorders.

## Simple Summary

Hepatitis C is widely known as a liver disease, but it can also affect the immune system and contribute to the development of disorders caused by the abnormal growth of certain white blood cells. These conditions include mixed cryoglobulinemia, a disorder that can damage blood vessels and organs, and some forms of lymphoma, a cancer of the immune system. Growing evidence shows that long-term infection with the hepatitis C virus plays a direct role in the development of these diseases. The introduction of modern antiviral treatments has dramatically improved the ability to eliminate the virus, with high cure rates and few side effects. Clearing the infection often leads to improvement of immune-related complications and can even cause some slow-growing lymphomas to regress. These findings support early treatment of hepatitis C and highlight the importance of integrating antiviral therapy into the care of patients with hepatitis C-related blood and immune disorders.

## 1. Introduction

Hepatitis C virus (HCV) infection affects approximately 50 million individuals worldwide and continues to represent a major global health burden [1]. HCV primarily causes chronic liver disease which, in a substantial proportion of patients, may progress to cirrhosis and hepatocellular carcinoma [2]. Beyond its hepatotropic nature, HCV is also a lymphotropic virus and is strongly associated with a wide spectrum of extrahepatic manifestations [3,4]. Among these, mixed cryoglobulinemia (MC) and B-cell non-Hodgkin lymphomas (B-NHLs)—including marginal zone lymphoma (MZL), lymphoplasmacytic lymphoma, and aggressive subtypes such as diffuse large B-cell lymphoma (DLBCL)—are the most clinically relevant, occurring in up to 20% of infected patients [5].

MC represents the most common extrahepatic manifestation of HCV infection and can be detected as a laboratory finding in 40–50% of cases, whereas clinical manifestations of cryoglobulinemic vasculitis (CV), historically also referred to as mixed cryoglobulinemic syndrome, occur in approximately 5–10% of patients. MC is currently regarded as a pre-lymphomatous B-cell lymphoproliferative disorder that may evolve into overt lymphoma in about 8% of cases [5]. Nevertheless, HCV infection may also be associated with the development of B-NHL even in the absence of detectable cryoglobulinemia [5].

A substantial body of epidemiological, clinical, and molecular evidence supports a direct pathogenic role of HCV in the development of both MC and B-NHL. Disease evolution from benign cryoglobulinemic disorders to overt B-NHL is thought to result from a complex, multistep pathogenic process [5]. A key mechanism is likely represented by the interaction between the viral envelope glycoprotein E2 and the CD81 receptor on B lymphocytes, which induces significant alterations in B-cell homeostasis, promoting chronic antigenic stimulation, clonal expansion, and progressive lymphoproliferation [6]. Notably, the presence of MC itself constitutes a major risk factor for the subsequent development of B-NHL [7].

Historically, antiviral therapy based on pegylated interferon (Peg-IFN) in combination with ribavirin (RBV), through viral eradication, demonstrated efficacy in the treatment of HCV-related MC [8,9]. Moreover, several studies reported that Peg-IFN–based regimens were capable of inducing HCV clearance along with regression of indolent, low-grade B-NHL, particularly MZL [10,11]. However, these therapeutic approaches were limited by suboptimal tolerability, high relapse rates, and significant toxicity, including neuropsychiatric adverse events and myelosuppression [10,11].

The advent of direct-acting antiviral agents (DAAs) has dramatically transformed the therapeutic landscape of HCV infection. Recent studies have shown that DAA therapy achieves sustained virologic response (SVR) in approximately 90–100% of patients, with no significant differences in SVR rates between patients with HCV-related MC and those without MC [12,13,14]. In addition, both clinical and immunological responses have been consistently observed in patients with HCV-related cryoglobulinemic vasculitis who achieve SVR following DAA therapy [12,13,14].

Emerging evidence further suggests that DAA-induced viral eradication, together with regression of MC, may be associated with complete or partial hematologic responses in HCV-related indolent lymphomas, particularly MZL [15,16,17,18]. Recent studies have shown that, despite successful viral eradication, a subset of patients continues to exhibit persistent B-cell clonal expansions, circulating rheumatoid factor-producing B-cell clones, and other signs of incomplete immunological recovery. These findings suggest that HCV-associated lymphoproliferative disorders may not be fully reversible following sustained virologic response. Furthermore, they have renewed interest in the pathogenic mechanisms linking chronic HCV infection to persistent B-cell dysregulation, immune system alterations, and the long-term risk of lymphomagenesis [19]. Although these findings are highly promising, confirmation in larger prospective studies with longer follow-up is still required [16,20]. Furthermore, preliminary data indicate that the concomitant use of DAAs and immunochemotherapy may improve outcomes in patients with HCV-associated DLBCL [15,21]. In this review, we summarize and critically evaluate the current evidence on HCV-related mixed cryoglobulinemia and B-cell lymphomas, with particular emphasis on the evolving role of direct-acting antivirals in the management of both indolent and aggressive lymphoid malignancies. We aim to provide a comprehensive overview of the impact of antiviral therapy on HCV-associated cryoglobulinemia and lymphoproliferative disorders by integrating virological, immunological, molecular, and clinical evidence. Owing to the substantial heterogeneity among available studies in terms of patient populations, lymphoma subtypes, treatment strategies, outcome measures, and follow-up duration, a formal meta-analysis was not feasible. In addition, many studies did not consistently report confidence intervals or comparable statistical estimates, limiting the possibility of quantitative synthesis. Therefore, this review presents a qualitative and critical appraisal of the currently available evidence and highlights areas requiring further investigation.

## 2. Methods

This review was conducted as a narrative review with a structured literature search. A comprehensive search of PubMed was performed to identify studies evaluating the relationship between HCV infection, mixed cryoglobulinemia, B-cell non-Hodgkin lymphoma, and the impact of DAA therapy. Original articles, prospective and retrospective cohort studies, clinical trials, and relevant review articles published in English were considered.

Studies were selected based on their relevance to the pathogenesis, clinical manifestations, and treatment outcomes of HCV-associated cryoglobulinemia and lymphoproliferative disorders. Data extraction included study design, patient characteristics, lymphoma subtype, antiviral treatment regimen, sustained virologic response, hematologic response, clinical response, and duration of follow-up.

The quality of the evidence was assessed descriptively by considering study design, sample size, duration of follow-up, completeness of outcome reporting, and potential sources of bias, including selection bias, retrospective data collection, and the absence of contemporaneous control groups.

Given the marked heterogeneity among studies regarding patient populations, lymphoma histology, therapeutic approaches, outcome definitions, and follow-up periods, a formal meta-analysis was not performed. Instead, findings were synthesized qualitatively and critically compared across studies. Numerical estimates derived from previously published meta-analyses are explicitly identified as pooled estimates, whereas percentages reported in Table 1, Table 2 and Table 3, represent ranges observed across individual studies and should not be interpreted as pooled measures of effect.

## 3. HCV-Related Cryoglobulinemia

Following the identification of HCV, it became evident that 90–95% of patients previously diagnosed with MC were actually infected with HCV [19,22]. This finding established HCV as the leading etiological factor in MC and significantly reshaped its clinical classification and management.

MC is classified into type II, characterized by monoclonal IgM with rheumatoid factor activity and polyclonal IgG, and type III, consisting of polyclonal IgM and IgG. HCV infection accounts for approximately 80–90% of MC cases, whereas a smaller proportion is associated with hepatitis B virus (HBV) infection, autoimmune disorders such as Sjögren’s syndrome, systemic lupus erythematosus, and rheumatoid arthritis (5–10%) [23]. In a minority of patients (5–10%), no underlying cause can be identified, and the condition is therefore classified as essential cryoglobulinemia [5,23,24].

In HCV infection, cryoglobulins are detectable in approximately 40–60% of patients; however, only a minority (about 5–30%) develop clinically overt cryoglobulinemic vasculitis (CV), the symptomatic clinical manifestation of MC [19]. Most patients (70–80%) develop mild to moderate CV, characterized by palpable purpura, asthenia, arthralgia, and sensory polyneuropathy, whereas 20–30% experience severe disease with skin ulcers, sensorimotor neuropathy, and major organ involvement [19,25].

Renal involvement occurs in nearly one-third of patients and most commonly manifests as membranoproliferative glomerulonephritis, typically presenting with proteinuria, hematuria, hypertension, and progressive renal dysfunction [5,19]. In rare cases (approximately 2–3%), CV may lead to life-threatening complications, including rapidly progressive glomerulonephritis, hyperviscosity syndrome, intestinal ischemia, alveolar hemorrhage, and central nervous system involvement [5,19].

Importantly, HCV-related MC has been strongly associated with the development of B-cell non-Hodgkin lymphomas, particularly indolent subtypes such as marginal zone lymphoma (MZL) and lymphoplasmacytic lymphoma, as well as aggressive forms including diffuse large B-cell lymphoma [19,26].

## 4. HCV-Associated Lymphoma

The association between HCV infection and B-NHL was first reported in 1994 [27], and has since been consistently confirmed by subsequent epidemiological studies and meta-analyses [28]. A meta-analysis including 15 case–control studies and 3 prospective studies reported a pooled relative risk of B- NHL of 2.5 (95% CI 2.1–3.0) in patients with HCV infection, with a higher risk observed in geographic areas with high HCV prevalence [26,29]. Moreover, the prevalence of B-NHL among HCV-infected patients has been shown to be higher than that observed in the general population (approximately 1.5%) and in patients with other hematologic malignancies (2.9%) [30].

Several distinct histological subtypes of B-NHL have been associated with HCV infection. The strongest associations have been reported for indolent lymphomas, particularly marginal zone lymphoma (MZL)—including splenic and extranodal forms—lymphoplasmacytic lymphoma, and, less frequently, aggressive lymphomas such as diffuse large B-cell lymphoma (DLBCL), which may arise de novo or through transformation from an underlying indolent lymphoma, most commonly MZL [5,19,31].

The pathogenesis of HCV-related lymphoproliferation is thought to parallel other antigen-driven lymphomas, such as Helicobacter pylori–associated gastric MALT lymphoma, and is largely based on chronic antigenic stimulation. Persistent viral stimulation of B lymphocytes promotes clonal expansion and may ultimately lead to malignant transformation [32]. Recent single-cell and immunogenetic studies have further refined this pathogenic model. In particular, Young et al. [33] showed that rheumatoid factor-producing B-cell clones may undergo somatic hypermutation, antigen-driven selection, and progressive clonal expansion, ultimately generating high-affinity cryoglobulin-producing cells. Although multi-omics, single-cell, and spatial approaches have not yet been widely applied to HCV-associated lymphoproliferative disorders, they may help clarify how immune dysregulation and tissue microenvironments sustain pathogenic B-cell clones after viral eradication [34,35,36]. These findings complement, rather than challenge, the classical antigen-driven hypothesis by providing a mechanistic framework that explains how chronic viral stimulation can promote the transition from polyclonal B-cell activation to autonomous clonal expansions. This model may also account for the persistence of pathogenic B-cell clones and immunological abnormalities in a subset of patients despite successful viral eradication [33]. In this context, the presence of HCV-related cryoglobulinemia represents a major risk factor for the development of B-NHL, with an estimated risk up to 35-fold higher than that of the general population [7].

In these conditions, expanded B-cell clones typically express stereotyped B-cell receptors (BCRs) with rheumatoid factor (RF) activity and putative specificity for the HCV E2 envelope protein [37,38,39,40,41]. Although direct reactivity of BCRs cloned from HCV-associated B-NHL with E2 has not been consistently demonstrated [42], the frequent regression of MC and, less commonly, indolent B-NHL following antiviral therapy strongly supports a pathogenetic role for chronic antigenic stimulation by the virus. Within this framework, immunogenetic studies have shown that HCV-associated lymphoproliferative disorders display a restricted BCR repertoire. While the biased usage of IGHV1-69 has long been recognized [39,41], more recent analyses highlighted the contribution of stereotyped κ light chains belonging to the IGKV3 family. In particular, IGKV3-20/3D-20 light chains may confer structural homology with antibodies directed against the HCV E2 protein and have been associated with a higher likelihood of lymphoma regression after viral eradication [43]. This observation is clinically relevant because it provides indirect support for the concept that a subset of HCV-associated indolent lymphomas remains dependent on chronic antigenic stimulation. Accordingly, patients harboring stereotyped B-cell receptor configurations, such as IGKV3-20/IGKJ3 (formerly designated 3D-20), may be more likely to achieve hematologic responses following antiviral therapy alone. These findings further support the hypothesis that lymphomagenesis in at least a subset of HCV-associated indolent lymphomas is driven by persistent antigenic stimulation and may therefore remain susceptible to therapeutic strategies targeting the underlying viral infection [42,43]. Notably, none of the clinical studies included in this review systematically reported BCR immunogenetic features; therefore, correlations between stereotyped BCR configurations and hematologic response to antiviral therapy remain speculative and require prospective validation.

However, recent single-cell analyses suggest that pathogenic clones may primarily represent autoreactive rheumatoid factor (RF) B cells recognizing IgG rather than viral antigens and progressively accumulating somatic mutations, including occasional lymphoma driver mutations, during chronic immune stimulation [33]. In this context, immune complexes containing immunoglobulin G (IgG) may contribute to the activation and persistence of rheumatoid factor (RF)-expressing B-cell clones. Experimental studies have shown that IgG-containing immune complexes can cooperate with innate immune stimuli to restore the proliferative capacity of otherwise exhausted RF-specific CD21^low^ B cells, supporting their role in sustaining pathogenic B-cell expansion even after viral eradication [44]. A possible integration of these two models could rely on the idea that Chronic HCV infection may initially select B-cell clones through recognition of viral antigens and viral-associated immune complexes. During prolonged stimulation, these clones may undergo affinity maturation and progressively acquire rheumatoid factor activity, eventually becoming predominantly autoreactive and partially independent of direct viral antigen recognition. Indeed, recent immunogenetic and single-cell analyses provide mechanistic insights into how chronic antigenic stimulation may progressively drive clonal selection, somatic hypermutation, and the emergence of persistent pathogenic B-cell populations, thereby linking long-term immune activation to lymphoproliferative disease. Taken together, these findings support a dynamic model of HCV-associated lymphoproliferation in which chronic antigenic stimulation by the virus initiates B-cell expansion, whereas immune complex-mediated signaling, ongoing clonal selection, somatic evolution, and the accumulation of additional molecular lesions promote the persistence and progression of pathogenic B-cell clones over time [37].

These findings may also help explain why, despite viral eradication with DAAs, clonal B-cell expansions can persist in a subset of patients, suggesting that additional antigen-independent or immune complex–mediated mechanisms may contribute to the maintenance of the lymphoproliferative clone [45].

From a clinical perspective, HCV-positive B-cell lymphomas are frequently characterized by splenomegaly and a higher rate of extranodal involvement, particularly affecting the spleen, liver, and salivary glands. Several studies have reported poorer outcomes in HCV-positive B-NHL patients, including higher mortality rates compared with HCV-negative counterparts. Furthermore, histologic transformation from indolent lymphoma to DLBCL has been associated with a more aggressive clinical course and worse prognosis [46].

## 5. Therapeutic Strategy

### Treatment of HCV-Related Mixed Cryoglobulinemia

The recognition of the causal relationship between HCV infection and MC has profoundly changed the therapeutic approach, establishing antiviral therapy as the cornerstone and first-line treatment. Historically, IFN-based regimens combined with ribavirin achieved viral eradication and clinical improvement in approximately half of patients; however, their use was limited by frequent relapses, poor tolerability, and significant adverse effects [19,47].

The clinical course of CV is closely linked to viral clearance, defined as sustained virologic response (SVR). The advent of direct-acting antiviral agents (DAAs) has revolutionized the management of chronic HCV infection and its extrahepatic manifestations [48,49]. DAA therapy achieves SVR rates of approximately 90–100% in patients with chronic HCV infection, including those with HCV-related MC [12,13,19,50].

DAA therapy is associated with high rates of clinical and immunological response, excellent tolerability, and a favorable safety profile. Across the individual studies summarized in Table 1, sustained virologic response rates ranged from 74% to 100%; however, these values should be interpreted descriptively because of substantial differences in study design, patient populations, treatment regimens, outcome definitions, and duration of follow-up [12,13,14,51,52,53,54,55,56,57,58,59,60,61,62,63]. Similarly, overall clinical response ranged from 60% to 97%, whereas immunological response ranged from 29% to 87%. These percentages reflect the variability of the individual studies and should not be interpreted as pooled estimates. Improvement in clinical manifestations and quality of life is frequently observed early during treatment [5,19,24,64,65,66], with further progressive improvement during follow-up reported in approximately half of cases [57]. Despite the high rates of sustained virologic response, some patients continue to experience persistent or recurrent vasculitic manifestations during follow-up. Reported relapse rates varied considerably across studies, reflecting differences in patient populations, disease severity, outcome definitions, and follow-up duration [13,67,68,69].

**Table 1:** Main studies reporting the outcome of DAA-based therapy in patients with HCV-related cryoglobulinemic vasculitis.

First Author, Year, (Ref.)	Number of Pts	SVR (%)	Overall CR (%)	Overall IR (%)	CV Relapse (%)	Post-Therapy FU (Weeks)
Saadoun, 2017 [51]	24	74	89	46	17	12
Sise, 2016 [52]	12	83	89	44	16	12–24
Gragnani, 2016 [12]	44	100	89	73	ND	24
Lauletta, 2017 [53]	22	100	86	73	14	12
Emery, 2017 [54]	18	89	62	29	29	20
Gragnani, 2018 [55]	85	100	97	87	ND	65
Mazzaro, 2018 [56]	22	95	75	5	5	48
Bonacci, 2018 [58]	46	100	91	48	11	96
Passerini, 2018 [57]	35	100	66	68	ND	ND
Cacoub, 2019 [14]	148	97	95	53	0	61
Pozzato, 2020 [59]	67	95	60	60	ND	96
Kondili, 2022 [13]	523	100	88	ND	13	96
Ferri, 2026 [60]	161	100	74	75	ND	291

Legend: Ref.: reference number; SVR: sustained virological response; overall CR: overall clinical response (complete + partial response); overall IR: overall immunological response (complete + partial response); CV: cryoglobulinemic vasculitis; FU: follow-up; ND: not determined. Note that the numerical values summarized in Table 1 are presented descriptively to illustrate the variability of outcomes across studies and should not be interpreted as pooled estimates or direct statistical comparisons.

Among the various clinical manifestations, cutaneous purpura—particularly of the lower limbs—typically shows the most rapid and pronounced response after viral eradication, whereas arthralgia and sicca symptoms tend to improve less consistently [12,14,51,52,53,54,55,56,58,59]. Low- to moderate-dose glucocorticoids and colchicine are often employed to control persistent purpura and arthralgia [67]. Sensory peripheral neuropathy improves in approximately 40% of patients, while severe motor neuropathy rarely responds to antiviral therapy alone [12,14,51,52,53,54,55,56,58,59]. Renal involvement, most commonly in the form of membranoproliferative glomerulonephritis, shows partial improvement in serum creatinine and proteinuria in about half of patients during follow-up [67].

In patients with severe or persistent manifestations—such as skin ulcers, significant renal involvement, or motor neuropathy—second-line therapy with rituximab (375 mg/m^2^ weekly for four weeks) after viral eradication has demonstrated additional clinical and immunological benefit [12,13,24,52,53,70]. In very severe or life-threatening cases, including rapidly progressive glomerulonephritis or major gastrointestinal, central nervous system, or pulmonary involvement, more aggressive therapeutic strategies such as plasma exchange and/or cyclophosphamide may be required [19,67]. From an immunological perspective, DAA therapy leads to a reduction in cryocrit in the vast majority of patients, although complete disappearance of circulating cryoglobulins is observed in only about 30% of cases. Rheumatoid factor levels normalize in approximately one-quarter of patients, and C4 levels often increase, although full normalization is less frequent [67]. Viral eradication is also associated with partial regression of B-cell clonal expansion, including the disappearance of detectable t(14;18) translocation in circulating B cells and a partial restoration of immune homeostasis among B- and T-cell subsets [19]. However, persistent and uncontrolled B-cell proliferation may still predispose to the development of overt lymphoma. The persistence of rheumatoid factor-producing clonal B cells despite sustained virologic response may help explain why some patients continue to experience residual cryoglobulinemic manifestations or remain at risk of subsequent lymphoproliferative complications. These observations suggest that, although viral eradication is essential for disease control, it may not always be sufficient to achieve complete immunologic remission. This appears to be particularly relevant in patients with long-standing disease, established clonal B-cell expansions, or advanced stages of clonal evolution, in whom pathogenic B-cell populations may persist independently of the original viral trigger. In addition, data of Young et al. [33] support the idea that viral eradication alone may not be sufficient to achieve complete immunologic remission in all patients. Persistent clonal B-cell populations may contribute to the heterogeneity of hematologic responses after DAA therapy and justify prolonged clinical surveillance, with B-cell-directed therapies considered in selected cases.

Overall, DAA therapy is highly effective in patients with mild-to-moderate cryoglobulinemic vasculitis, particularly for purpura, asthenia, arthralgia, and sensory neuropathy. However, in patients with severe organ involvement, antiviral therapy alone may be insufficient, and adjunctive treatment with rituximab or other anti-CD20 monoclonal antibodies is often required [25]. Notably, rituximab administered after antiviral therapy has been associated with significant improvement in severe cryoglobulinemic vasculitis, even in patients with underlying HCV-related cirrhosis [25]. Despite the high efficacy of DAAs, relapses of purpura, arthralgia, or severe vasculitis during follow-up have been reported in a substantial proportion of patients. However, the frequency of relapse varied considerably across the available studies (Table 1), although direct comparison is limited by substantial methodological heterogeneity [12,13,14,51,52,53,54,55,56,57,58,60,67].

## 6. Therapy of HCV-Associated B-NHL

### Antiviral Therapy with DAAs in HCV-Associated B-NHL

The therapeutic benefit of HCV eradication in patients with B-NHL was first demonstrated in the era of pegylated interferon (Peg-IFN) plus ribavirin therapy. In these early studies, virologic response rates ranged from 50% to 76% and were associated with hematologic responses in approximately half of cases [10,11]. This observation was conceptually analogous to the regression of gastric MALT lymphoma following Helicobacter pylori eradication, supporting the role of chronic antigenic stimulation in lymphomagenesis. However, interferon-based regimens were limited by significant toxicity, including neutropenia, thrombocytopenia, ribavirin-induced hemolytic anemia, frequent relapse of vasculitis, and overall poor tolerability [10].

As previously discussed, DAAs achieve very high rates of SVR and are currently recommended by international guidelines for the treatment of HCV infection and its extrahepatic manifestations, including B-NHL [19,47,70].

Several retrospective and prospective studies (Table 2) have evaluated the role of DAAs in patients with HCV-associated indolent, low-grade B-NHL, particularly marginal zone lymphoma (MZL) [20,71,72,73,74]. In many of these studies, regression of lymphoma has been observed following viral eradication, further supporting the etiological role of chronic HCV-driven antigenic stimulation [16,20,71,72,73]. Arcaini et al. [16] reported a hematologic response rate of 67% in a cohort of 46 patients with indolent B-NHL treated with DAAs, with 98% achieving SVR. Similarly, Frigeni et al. described an overall response rate of 66% in 66 patients, including complete response in 23% and partial response in 43% of cases [72]. Notably, in some series, no significant hematologic response was observed in patients with chronic lymphocytic leukemia, suggesting a more limited role of viral eradication in this subtype [16,67].

**Table 2:** Main studies assessing the effects of DAA-based therapy in patients with HCV-related low-grade non-Hodgkin’s lymphoma.

First Author, Year, (Ref.)	Number of Pts	B-NHL Type (%)	MC (%)	SVR (%)	B-NHL Hematologic Response (%)	MC Clinical Response (%)	FU Months
Carrier, 2015 [20]	3	MZL 3 (100)	3 (100)	3 (100)	CR 3 (100)	3 (100)	9–12
Alric, 2016 [71]	7	MZL 7 (100)	7 (100)	7 (100)	CR 9 (90) PR 1 (10)		12
Arcaini, 2016 [16]	46	MZL 37 (80), Non-MZL 9 (20)	15 (33)	46 (100)	CR 12 (26) PR 19 (41) SD 11 (24) NR 4 (9)	7 (47)	8
Frigeni, 2020 [72]	66	MZL 53 (80) Non-MZL13 (20)		66 (100)	CR 14 (21) PR 31 (47) SD 15 (23) NR 5 (8)		17
Merli, 2022 [73]	40	MZL 27 (67%) Non-MZL 13 (33%)	14 (35)	40 (100)	CR 8 (20) PR 10 (25) SD 16 (40) PD 6 (15)	8 (57)	37

Legend: Ref.: reference number; B-NHL: Non-Hodgkin’s lymphoma; MC: Mixed Cryoglobulinemia; SVR: sustained virological response; FU: follow-up; MZL: marginal zone lymphoma; Non-MZL: non-marginal zone lymphoma; CR: complete response; PR: partial response; SD: stable disease; NR: no response.

More recently, a prospective study by Merli et al. [73] which included 40 patients with HCV-associated indolent non-Hodgkin lymphoma, reported a sustained virologic response rate of 100% and an overall hematologic response rate of 45% after a median follow-up of 37 months. These findings are consistent with the potential efficacy of DAA therapy in selected patients with indolent lymphoma, particularly those with marginal zone lymphoma, although interpretation is limited by the observational design and heterogeneity of the available studies. Collectively, the available evidence suggests that antiviral therapy may represent an important component of an individualized treatment strategy, although the available studies are heterogeneous and largely observational.

Nevertheless, hematologic response rates observed with DAAs appear somewhat lower than those historically reported with IFN-based therapy. This difference may be explained by the direct antiproliferative and immunomodulatory effects of IFN-α on the malignant B-cell clone, in addition to its indirect antiviral activity [67].

Our recent study [75] further supports this concept. In a cohort of 23 patients with HCV infection and low-grade B-NHL or monoclonal B-cell lymphocytosis treated with DAAs, SVR was achieved in all cases and a clear clinical improvement of CV was observed. However, B-cell clonal expansion persisted in the majority of patients after antiviral therapy, with disappearance of the malignant clone documented in only 11% of cases, both of whom had received additional lymphoma-directed treatment. The persistence of clonal B-cell populations after viral eradication is consistent with recent single-cell analyses demonstrating ongoing clonal evolution and acquisition of additional molecular abnormalities within pathogenic RF-producing B-cell clones [33]. These findings suggest that responsiveness to antiviral therapy may vary according to the biological stage of clonal evolution, with earlier antigen-dependent lesions being more likely to regress after HCV eradication than more autonomous lymphoproliferative clones.

In aggressive lymphomas, such as DLBCL, DAAs have mainly been administered concomitantly with chemo-immunotherapy rather than as standard lone therapy. A retrospective study by Persico et al. [21] reported a complete response rate of approximately 90% in 20 patients treated with DAAs in combination with chemotherapy, with outcomes comparable to historical controls. Similarly, Merli et al. [15] described a cohort of 47 patients receiving DAAs during chemotherapy, reporting a complete response rate of 98%, a 2-year progression-free survival of 93%, and an SVR rate of 98%, with good tolerability and reduced hepatic toxicity. Comparable results were also reported by Occhipinti et al. [76] (Table 3).

**Table 3:** Main studies assessing the effects of DAA-based therapy in patients with HCV-related high-grade non-Hodgkin’s lymphoma.

First Author, Year, (Ref.)	Number of Pts	B-NHL Type (%)	MC (%)	SVR (%)	Chemo-Therapy (%)	B-NHL Response (%)	FU Months
Carrier, 2015 [20]	2	DLBCL 2 (100)		2 (100)	2 (100)	CR 2 (100)	12
Alric, 2016 [71]	3	DLBCL 3 (100)		3 (100)	3 (100)	CR 2 (100)	12
Persico, 2018 [21]	20	DLBCL 20 (100)		20 (100)	20 (100)	CR 19 (95)	8
Occhipinti, 2019 [76]	7	DBLCL 7 (100)		7 (100)	7 (100)	CR 7 (100)	12
Merli, 2019 [15]	47	DLBCL 45 (96) FL 2 (4)	5 (11)	45 (96)	47 (100)	CR 46 (98)	33.6

Legend: Ref: reference number; B-NHL: non-Hodgkin’s lymphoma; MC: Mixed Cryoglobulinemia; SVR: sustained virological response; FU: follow-up; DLBCL: diffuse large B-cell lymphoma; FL: follicular lymphoma; CR: complete response; PD: progressive disease; MC: mixed cryoglobulinemia; PR: partial response.

The interpretation of these findings is limited by several methodological constraints, including the retrospective design of most studies, small sample sizes, potential selection bias, and reliance on historical control groups that may not fully account for temporal advances in supportive care, lymphoma management, and antiviral therapy. Consequently, although the available data are encouraging, the specific contribution of direct-acting antivirals to improved clinical outcomes cannot be definitively established. Prospective studies with appropriate contemporary control groups are needed to better define the impact of antiviral therapy in this setting.

To date, no prospective clinical studies have specifically evaluated DAA therapy alone in patients with high-grade HCV-associated lymphomas. Current guidelines recommend initiating antiviral therapy either before or concomitantly with chemotherapy in these patients. Given the potential for drug–drug interactions, management requires a multidisciplinary approach and careful therapeutic monitoring [70]. Particular attention should be paid to the timing of antiviral therapy in relation to immunochemotherapy, potential drug–drug interactions between direct-acting antivirals and antineoplastic agents, careful monitoring of liver function, and the risk of hepatotoxicity during lymphoma treatment [19]. Close collaboration between hepatologists and hematologists is therefore essential to optimize treatment sequencing, ensure appropriate monitoring, and minimize treatment-related complications.

Importantly, antiviral therapy may also have a preventive role in lymphomagenesis. Mahale et al. demonstrated that the incidence of lymphoma in HCV-infected patients was lower after antiviral treatment compared with untreated individuals, suggesting that early viral eradication may reduce the risk of B-NHL development [77].

Overall, these findings indicate that DAA-based antiviral therapy plays a central role in the management of HCV-associated indolent B-NHL and represents an essential adjunctive strategy in high-grade lymphomas treated with chemo-immunotherapy, further reinforcing the pathogenetic role of HCV in B-cell lymphoproliferative disorders.

## 7. Conclusions

A substantial body of epidemiological, pathological, and therapeutic evidence supports a strong association between HCV infection and the development of lymphoproliferative disorders, particularly mixed cryoglobulinemia and B-NHL. Although this relationship is now well established, the precise mechanisms through which chronic HCV-driven immune stimulation and B-cell activation evolve into overt lymphoma remain only partially understood and require further elucidation.

Recent advances in immunogenetic and single-cell analyses have also provided new insights into the pathogenesis of HCV-associated lymphoproliferative disorders. These studies indicate that expanded B-cell clones frequently express rheumatoid factor–like B-cell receptors and may be sustained not only by viral antigens but also by IgG-containing immune complexes that promote the persistence of autoreactive B-cell populations. These mechanisms may help explain why clonal B-cell expansions can persist in some patients despite viral eradication.

The efficacy of DAAs in the treatment of HCV-associated CV is firmly established. DAA therapy is currently recommended as the first-line approach owing to its high SVR rates, short treatment duration, and favorable safety and tolerability profile. Viral eradication is closely associated with significant clinical and immunological improvement, although complete remission of vasculitis is not achieved in all patients and relapses may occur during long-term follow-up.

Encouraging outcomes have also been reported in patients with HCV-associated indolent, low-grade B-NHL. In this setting, antiviral therapy could be considered an appropriate initial therapeutic strategy in selected patients who do not require immediate lymphoma-directed treatment. HCV eradication may confer dual benefits, contributing both to the control of hepatic disease and to lymphoma regression in a subset of cases. Moreover, emerging evidence suggests that early antiviral treatment may play a preventive role in lymphomagenesis, potentially reducing the risk of B-NHL development, although this hypothesis requires confirmation in larger prospective studies with long-term follow-up.

In patients with HCV-positive high-grade lymphomas, the role of DAAs has been explored in a limited number of studies, mainly in combination with immunochemotherapy. Available data indicate that antiviral therapy administered before or concomitantly with chemotherapy is safe, improves hepatic tolerance to oncologic treatment, and may reduce the risk of HCV reactivation during therapy.

Only a small proportion of patients fail to achieve a sustained virologic response or experience virologic relapse following direct-acting antiviral therapy. Current treatment guidelines recommend retreatment with rescue antiviral regimens, typically incorporating agents with a high barrier to resistance. In patients with persistent viremia, ongoing viral antigenic stimulation may contribute to the persistence or recurrence of cryoglobulinemic manifestations and lymphoproliferative disorders. Accordingly, these patients require careful hepatologic reassessment and continued hematologic monitoring to evaluate disease activity, guide further therapeutic interventions, and detect potential progression of lymphoproliferative disease.

Overall, these findings support the integration of antiviral therapy into the multidisciplinary management of HCV-associated lymphoproliferative disorders, tailored according to lymphoma subtype, disease severity, and the need for immediate oncologic treatment.

## Data Availability

No new data were created or analyzed in this study. Data sharing is not applicable to this article.

## Abbreviations

HCV	Hepatitis C virus
MC	Mixed cryoglobulinemia
CV	Cryoglobulinemic vasculitis
B-NHL	B-cell non-Hodgkin lymphoma
DAAs	Direct-acting antivirals
SVR	Sustained virologic response
MZL	Marginal zone lymphoma
DLBCL	Diffuse large B-cell lymphoma
